# APOε2 and education in cognitively normal older subjects with high levels of AD pathology at autopsy: findings from the Nun Study

**DOI:** 10.18632/oncotarget.4118

**Published:** 2015-05-12

**Authors:** Diego Iacono, Peter Zandi, Myron Gross, William R. Markesbery, Olga Pletnikova, Gay Rudow, Juan C. Troncoso

**Affiliations:** ^1^ Neuropathology Division, Department of Pathology, Johns Hopkins University, Baltimore, MD, USA; ^2^ Neuropathology Research, Biomedical Research Institute of New Jersey, Cedar Knolls, NJ, USA; ^3^ Atlantic Neuroscience Institute, Overlook Medical Center, Summit, NJ, USA; ^4^ Department of Neurology, Icahn School of Medicine Mount Sinai, New York, NY, USA; ^5^ Department of Mental Health, Johns Hopkins University, Baltimore, MD, USA; ^6^ Department of Laboratory Medicine and Pathology, University of Minnesota, Minneapolis, MN, USA; ^7^ Department of Pathology, Sanders-Brown Center on Aging, University of Kentucky, Lexington, KY, USA; ^8^ Department of Neurology, Sanders-Brown Center on Aging, University of Kentucky, Lexington, KY, USA; ^9^ Alzheimer's Disease Center, Sanders-Brown Center on Aging, University of Kentucky, Lexington, KY, USA; ^10^ Department of Neurology, Johns Hopkins University, Baltimore, MD, USA

**Keywords:** Gerotarget, AD pathology, APOε2, higher education and language skills, neuronal hypertrophy, preserved cognition

## Abstract

Asymptomatic Alzheimer's disease (ASYMAD) subjects are individuals characterized by preserved cognition before death despite substantial AD pathology at autopsy. ASYMAD subjects show comparable levels of AD pathology, i.e. β-amyloid neuritic plaques (Aβ-NP) and tau-neurofibrillary tangles (NFT), to those observed in mild cognitive impairment (MCI) and some definite AD cases. Previous clinicopathologic studies on ASYMAD subjects have shown specific phenomena of hypertrophy in the cell bodies, nuclei, and nucleoli of hippocampal pyramidal neurons and other cerebral areas. Since it is well established that the allele APOε4 is a major genetic risk factor for AD, we examined whether specific alleles of APOE could be associated with the different clinical outcomes between ASYMAD and MCI subjects despite equivalent AD pathology. A total of 523 brains from the Nun Study were screened for this investigation. The results showed higher APOε2 frequency (*p* < 0.001) in ASYMAD (19.2%) *vs*. MCI (0%) and vs. AD (4.7%). Furthermore, higher education in ASYMAD *vs*. MCI and AD (*p* < 0.05) was found. These novel autopsy-verified findings support the hypothesis of the beneficial effect of APOε2 and education, both which seem to act as contributing factors in delaying or forestalling the clinical manifestations of AD despite consistent levels of AD pathology.

## INTRODUCTION

Many decades of clinicopathologic investigations on sporadic Alzheimer's disease (AD) have enormously contributed to the definition of the main pathologic lesions associated with AD, i.e. *β*-amyloid neuritic plaques (A*β*-NP) and tau-neurofibrillary tangles (tau-NFT), and to a better understanding of the possible relationships between cognitive deficits and AD lesions [1, 2]. However, the exact mechanisms leading to those pathologic hallmarks of AD (A*β*-NP, tau-NFT) and how they determine neuronal degeneration in specific areas of human brain (i.e., entorhinal cortex, hippocampus) yet remain to be yet completely clarified [3-5]. The hypothesis that A*β*-NP, tau-NFT, as well as A*β* or tau oligomers, are the only responsible factors for the cognitive declining in AD has not been satisfactorily confirmed by a series of neuropathologic, neuroimaging, and biochemical studies attempting to establish linear correlations between AD pathology and cognitive decline [6-12]. One of the most convincing evidences of the absence of linearity between cognitive deficits and AD pathology is the frequent observation, at autopsy, of a considerable number of older individuals with high burdens of A*β*-NP and tau-NFT with preserved cognition as assessed shortly prior to death (i.e. < 1 year). These older individuals with preserved cognition and consistent amounts of AD pathology at autopsy have been termed asymptomatic AD (ASYMAD) subjects. ASYMAD subjects not only have comparable levels of AD pathology to those found in mild cognitive impairment (MCI) subjects and some definite AD patients but also have an identical cerebral localization [13, 15]. Other investigators referred to ASYMAD under different appellations, such as “high pathology controls” [16] or “preclinical AD” [17]. Those investigators essentially referred to the same concept: there are older individuals cognitively silent for AD even with consistent levels of AD pathology at autopsy. This clinicopathologic dissociation has also been described employing “*in vivo*” functional neuroimaging studies [18-22].

The reason to term older individuals cognitively preserved but positive for AD pathology at autopsy using the acronym ASYMAD is because it is virtually impossible to know “*a priori”* whether these subjects would have remained cognitively preserved or eventually would have progressed to MCI or AD had they lived longer. The term ASYMAD then has the advantage to not imply necessarily a forthcoming or future cognitive decline [23]. In addition, the ASYMAD term can be easily applied to other fields of AD and aging research, such as functional neuroimaging [24].

It is significant to recall here that a clinicopathologic dissociation between AD pathology and cognition was already recognized a few decades ago [25-31] without receiving, unfortunately, any major scientific attention. Recently however, the discrepancy between cognitive aspects and AD pathology has been implicitly accepted by newer AD pathologic criteria [32, 33]. These criteria do not require a diagnosis of dementia to stage the AD pathology at autopsy. They “simply” propose a probabilistic scoring system for the AD neuropathologic changes, which uses previously established systems of A*β*-NP and NFT staging without taking into account any clinical or dementia history of the subject that received the autopsy.

In previous studies [34-37], a marked neuronal hypertrophy in different cerebral regions of ASYMAD subjects vs. age-matched controls (C), MCI and AD subjects was measured. These cellular changes have been hypothesized to be possible neuronal reactions or part of compensatory mechanisms facing the accumulation of AD pathology, which would allow normal cognitive functioning despite abundant AD pathology. In agreement with this compensatory hypothesis, phenomena of neuroplasticity have been described by functional neuroimaging studies enrolling MCI and early-AD patients and also, importantly, high-risk subjects for AD, that is subjects without cognitive signs of the disease [37-41].

This new clinicopathologic investigation aimed to go beyond the morphometric characterizations [35, 36] and quantitative measurements of AD pathology in ASYMAD vs. Controls, MCI, and definite AD [42] and possibly describe other contributing factors which could underlie the cognitive resilience of ASYMAD subjects despite AD pathology.

There are very few large epidemiological studies that include analyses of normal aging and AD, and an autopsy program. Among those rarer epidemiological studies, the Nun Study has been a historical one [43]. Rendering use of the wealth of longitudinal clinical information and rigorous pathological observations available from the Nun Study [43, 44], we aimed to analyze the allelic frequencies of APOE gene (APOE) and the attained education levels of the entire available sample of this unique autopsy-cohort. APOε2, one of the three alleles of APOE [45], and higher education levels have been shown to have protective capacities against AD [46, 47]. The principal aim and novelty of this investigation was to verify if APOε2 frequency and education levels were indeed significantly higher in ASYMAD subjects from the Nun Study. An adjunctive potential novelty of this study consisted in the opportunity to associate those possible findings on the protective effects of APOε2 and higher education taking also into account previously described phenomena of neuronal hypertrophy in ASYMAD subjects from the same study [37], as well as their association to higher language skills acquired early in life. Higher early-acquired language skills have been demonstrated to reduce the risk of dementia [48-49].

## RESULTS

Demographic, educational, cognitive, neuropathologic, and APOE allele distribution data across groups are summarized in Table 1. Statistical analyses did not show differences for the mean age at death, age at last cognitive assessment, and Cog-Death interval across all groups. BW was not different among ASYMAD, MCI, and C, although the mean BW was lower in AD compared with all other groups.

**Table 1:** Demographic, educational, cognitive, neuropathologic, and APOE frequencies of 155 subjects with autopsy-confirmed diagnosis and interval Cog-Death ≤1.0 year

	Controls (n = 11; 7.1%)	ASYMAD (n = 13; 8.3%)	MCI (n = 15; 9.6%)	AD (n = 116; 74.8%)
**Interval cog-death (years)**	0.5±0.2	0.50.2	0.5±0.2	0.40.2
**Age at death (years)**	86.9±5.7	89.5±2.1	89.4±4.5	92.2±4.6
Age at last cog (years)*	86.4±5.8	89.0±2.3	88.8±4.5	91.7±4.6
Last MMSE*	27.8±1.3	28.3±1.1	26.0±1.4	8.0±8.1
ADLs*	5.0±0.0	5.0±0.0	4.7±0.4	0.9±1.5
**Education (%)**				
** Grade school only**	0.0	7.6	6.6	16.3
** High school diploma**	0.0	0.0	0.0	7.7
** Bachelor degree**	36.6	30.7	60.0	46.5
** Masters or higher**	63.4	61.5	33.3	29.3
**Brain weight (grams)**	1182.2±259.3	1144.5±80.5	1170.3±110.9	1083.9±117.1
CERAD score (%)*				
** 0**	100	0.0	0.0	0.0
** B**	0	76.9	73.3	41.3
** C**	0	23.0	26.6	58.6
NFT Braak score (%)*				
** 0**	18.1	0.0	0.0	0.8
** I**	27.2	15.3	6.6	3.4
** II**	54.5	69.2	33.3	12.0
** III**	0.0	15.3	6.6	12.0
** IV**	0.0	0.0	20.0	11.2
** V**	0.0	0.0	33.3	22.4
** VI**	0.0	0.0	0.0	37.9
ε2 allele frequency (%)*	13.6	19.2	0	4.7
**ε3 allele frequency (%)**	86.3	76.9	70.0	75.8
ε4 allele frequency (%)*	0	3.8	30.0	19.4

* Differences across groups were significant at *p*<0.01. Controls: age-matched controls; ASYMAD: asymptomatic AD subjects; MCI: mild cognitive impairment subjects; AD: Alzheimer's disease patients. The interval Cog-Death (the interval of time between the last cognitive assessment and death), age at death, age at last cognitive exam, last MMSE, ADLs, and brain weight are expressed as mean±SD. The first row of the table shows the sample size (n and percentages) of each group respect to the total sample size (n = 155) of the study. The table shows also the single APOE allele (ε2, ε3, and ε4) frequencies for each group.

ASYMAD and C did not differ for the last MMSE and ADLs mean scores. By contrast, AD showed significantly lower MMSE and ADLs mean scores compared to all other groups.

A*β*-NP CERAD scores showed no statistical difference between ASYMAD and MCI, whereas differences were found between ASYMAD and AD (*p* < 0.05); and between MCI and AD (*p* < 0.05). Braak-NFT scores were different across comparisons when all stages (0-VI) were considered: ASYMAD vs AD (*p* < 0.001), MCI vs AD (*p* < 0.01), and ASYMAD vs MCI (*p* < 0.05). However, when the analysis of Braak staging was performed including only the first five stages (0-IV), no differences were observed between ASYMAD vs. MCI and even between MCI and AD.

**Figure 1:**
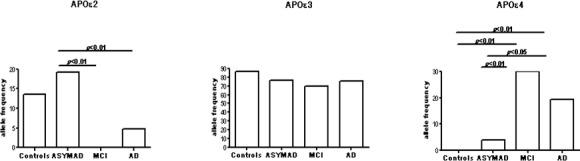
The figure shows histograms for the frequency distribution of APOε2, ε3, and ε4 alleles in the four different groups examined in the study

Higher APOε2 frequency was found in ASYMAD (19.7%) compared to MCI (0%, *p* < 0.01) and AD (4.7%, *p* < 0.01). No different APOε2 frequency was detected between ASYMAD and C (13.6%). While no differences were observed in APOε3 frequency across all groups, lower APOε4 frequency was observed in ASYMAD (3.8%) compared to MCI (30.0%, *p* < 0.01) and AD (19.4%, *p* < 0.01). Importantly, no different APOε4 frequency was found between ASYMAD and C (0%). All comparisons for APOE allelic frequencies are shown in Figure 1.

**Figure 2:**
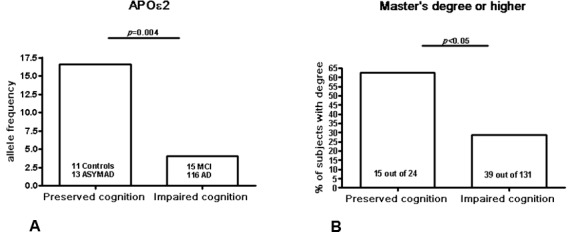
The figure shows the histograms for APOε2 frequencies and educational levels in two different groups: 1) preserved cognition group (PCG): controls+ASYMAD subjects; and 2) impaired cognition group (ICG): MCI+AD patients. The p-values are the statistical significance values after exact Fisher's test

Moreover, APOε2 frequency was significantly higher (*p* = 0.004) in PCG (C+ASYMAD, ε2 = 16.6%) compared to ICG (MCI+AD, ε2 = 4.2%) (Figure 2). By contrast, whereas APOε3 frequency did not show any difference between these two groups, APOε4 frequency was significantly higher (*p* = 0.001) in ICG (ε4 = 20.6%) compared to PCG (ε4 = 2.0%).

The analyses show significant differences on education levels between PCG compared to ICG (*p* = 0.02) group. Correlation analyses (Spearman's test) between APOε2 and the highest level of attained education (master or higher), however, did not show significant correlations across all groups, or any other possible correlation between groups comparison.

## DISCUSSION

The most important findings of this clinicopathologic study are: a) higher APOε2 frequency and b) higher educational levels in ASYMAD vs. MCI and AD. These findings suggest a beneficial effect of APOε2 and education on later cognition even in presence of AD pathology burdens that are equivalent to those found in the MCI and some definite AD cases. Previous epidemiological studies have reported a protective role of APOε2 [46] and higher education [60] against AD, but only few have been confirmed by autopsy findings [61]. To the best of our knowledge, this is the first clinicopathologic study showing a significant association between higher APOε2 frequency and higher education in a cohort of autopsy-confirmed ASYMAD vs. MCI, and AD subjects. Moreover, from the same autopsy-cohort, a previously examined group of ASYMAD subjects was associated with specific cellular morphometric changes, such as neuronal hypertrophy in CA1 of hippocampus and higher language skills acquired in early life [37]. Unfortunately, the limited availability of hippocampi from all autopsy cases of this study did not allow to verify those significant correlations among CA1-neuronal hypertrophy, APOε2, and education/language across the entire autopsy-cohort. However, this investigation seems to offer important seminal perspectives on the possible interaction among AD pathology, APOE genotypes, education/language, and mechanisms of neuroplasticity. These intriguing cognition-pathology-genotype-neuroplasticity correlations remain to be verified by future larger autopsy-studies. However, a recent non-autopsy study confirmed our previous findings [37] on the beneficial effect of APOε2 on lifetime experiences enhancing cognition, which was better indicated by measuring specific linguistic skills (i.e., vocabulary and reading ability) [62].

In terms of pathogenic sequence, our findings suggest that the possible beneficial effects of APOε2 and higher education lie downstream to both A*β* and tau depositions, and are possibly linked to multiple biological mechanisms [63-64]. A recent larger neuropathologic study [65], which analyzed a larger amount of pathologic and clinical data from the National Alzheimer's Coordinating Center (NACC) dataset, had the chance to stratify a higher number of subjects based on their levels of AD pathology and clinical manifestation. Their findings are in line with ours regarding the protective effect of APOε2 even after adjustments for influencing factors such as the age of onset, duration of symptoms, and other demographic variables. Although the nature of the population analyzed in this larger study [65] is different from ours, the findings are impressively similar. Moreover, our findings not only seem to confirm the results from a larger study [65] but also extend them. In fact, the novelty of our investigation consists not only in supporting that ASYMAD are subjects associated with higher APOε2 frequency and education but also with specific morphometric-neuronal changes [37] and cognitive skills, such as higher early-acquired language skills [37, 48, 49].

Moreover, experimental studies indicate that APOε2 has a protective role in both the peripheral and central nervous system [66, 67]. Furthermore, data describe APOε2's enhancing capacity to increase neuritic growth in contrast to APOε4 which demonstrates a detrimental effect on it [68, 69].

Remarkably, APOε3 frequency was not different across all groups in our investigation. This suggests that it is actually the interplay between APOε2 and APOε4 to weigh heavily on the risk of developing MCI and AD, or remaining cognitively unscathed. Importantly, only the presence of APOε2 is insufficient to avoid the AD pathology deposition, as demonstrated by the conspicuous amount of it in ASYMAD subjects. APOε2, rather, seems to have beneficial properties in reducing the consequences of AD pathology accumulation or even in reducing it [70].

APOE distribution in the Nun Study is similar to that of Caucasian populations [71]. However, it is important to emphasize that APOε2 frequency in this Nun Study autopsy-cohort was curiously higher (19.2%) than that observed in Caucasians (2-8%). This could be related to the possible role of APOε2 on survival and longevity [72-74]. The involvement of APOε4 as risk factor for cardiovascular diseases [75] and enhanced innate immune response [76], could exclude these subjects from normal aging and AD longitudinal studies.

How common is ASYMAD? In our series, out of 24 cognitively preserved subjects (C+ASYMAD) older than 85 years (mean age = 88.2±3.9), 54.0% showed severe levels of AD pathology but unimpaired cognition (ASYMAD). A previous large autopsy-cohort study [77] showed that 30% of subjects diagnosed with probable AD have insufficient AD neuropathological changes at autopsy to satisfy a diagnosis of definite AD. While those data showed insufficiency of AD pathology to explain the dementia in a high number of subjects, we substantiate that even with equivalent amounts of AD pathology, other factors such as APOε2, education, and early acquired language/cognitive skills [33] are indeed associated with a decreased risk, delay, or even avoidance of dementia.

It is not possible to transfer our findings to the general population without caveats. We cannot exclude that the higher educational levels of the Nun Study participants contributed to a higher “cognitive reserve” [78]. In the Nun Study though, this issue could be less of a challenge since all sisters shared the lifestyle and comparable medical care. Other caveats could be the exclusion of those factors that can also impair the cognition: cerebro-vascular hypoxic phenomena, metabolic disorders, diabetes, hyperlipidemia, untreated hypertension, hypercaloric diet, alcohol abuse, or environmental and lifestyle stressors. It is neither possible to exclude that two brains can have similar numbers of A*β*-NP and NFT, but one brain has a higher concentration of toxic and specific soluble A*β* or tau oligomers than the other [12], and its risk of functional impairment is therefore enhanced. Genetic factors different from APOE and non-genetic factors different from education could also mutually interact. Complex gene-environment interactions, in fact, seem to determine an increased or decreased risk of dementia later in life. Recent studies on twins describe, for example, that even identical twins can differ in their onset of dementia and have different amounts of AD pathology at autopsy [79].

## MATERIALS AND METHODS

Subjects were identified by querying the database of the Nun Study, which includes clinical and cognitive data, educational information, neuropathologic evaluations with definite diagnoses, and APOE genotypes. Participants in the Nun Study are Catholic sisters belonging to the School Sisters of Notre Dame congregation living in various communities in the Midwestern, eastern, and southern regions of the US [43]. At the time of the enrollment period all sisters born before 1917 were asked to participate in the study. Of 1027 eligible sisters (aged 75 years or older), 678 (66.0%) agreed to participate in the study. All sisters participating in the study signed an informed written consent form and agreed to annual physical and cognitive assessments and review of their medical records. They also consented to donate their brains for research after death. The Nun Study autopsy procedures were approved by the University of Kentucky's Institutional Review Board. In 2010, the Nun Study moved back to its original Institution, the University Of Minnesota.

In the present investigation only the participants that underwent autopsy were considered. The total sample size of the cohort with brain autopsy available was 523, that is, the 77.1% of the entire eligible cohort. The methods used to assess the cognitive status have been described in previous publications [50]. Briefly, the cognitive test battery included measures compiled by the Consortium to Establish a Registry for Alzheimer's disease (CERAD) [51], which assesses memory, language, visuo-spatial ability, concentration, and orientation. In the Nun Study, this neuropsychological battery was administered by two trained gerontologists. Five of the tests from the CERAD battery were used to define preserved cognitive function: Mini-Mental State Exam (MMSE), Delayed Word Recall, Boston Naming, Verbal Fluency, and Constructional Praxis. The standard cut point of 24 or greater was used to identify intact scores for the MMSE. For the other four tests, cut points that were close to, but did not exceed the 5th percentile for the normative data described by the CERAD group [52], were identified to classify individual test scores as intact or impaired. The cut points for intact scores were as follows: ≥13 for Boston Naming, ≥11 for Verbal Fluency, ≥4 for Delayed Word Recall, and ≥8 for Constructional Praxis. Participants judged to be cognitively intact in the present analyses had intact scores on all five tests. Individuals who were classified as demented in the study had each of the following conditions: (a) impairment in memory and in at least one other area of cognition, (b) impairment in social or daily function (i.e., inability to use a phone, handle money, or dress oneself), and (c) decline in function from a previous level (observed during our study for the incident dementia cases, or inferred for those dementia cases present at the first exam). From the above described battery, the following classification system was established to define a subject cognitively as:

Intact (Preserved) Cognition: intact scores on all cognitive tests and activities of daily living (ADL). This category defined the cognition level of both age-matched controls (C) and ASYMAD subjects.

Mildly Impaired Cognition (with memory impairment): intact on MMSE and ADL, but impaired on the Delayed Word Recall and one or more of the other three cognitive tests. This category is equivalent to mild cognitive impairment (MCI) subjects based on Mayo Clinic and AA-NIA MCI criteria [53, 54].

Demented: met clinical criteria for dementia as described above.

### Neuropathologic material and methods

The Nun Study had neuropathological data on a total of 523 autopsy brains. These brains were removed and examined macroscopically after fixation in 10% buffered formaldehyde for at least 2 weeks. Tissues blocks were dissected from middle frontal gyrus, superior and middle temporal gyri, inferior parietal lobule, occipital cortex (area 17-18), entorhinal cortex, hippocampus, amygdala, thalamus, basal ganglia and cerebellum. Tissue blocks were processed and embedded in paraffin, cut at 10 μm, and stained with hematoxylin-eosin (H&E) and Hirano-silver method. The severity of A*β*-NP was assigned by semi-quantitative and age-adjusted scores (0, A, B, or C) according to CERAD pathologic criteria [55], and NFT stages were scored (0-VI) according to Braak-NFT system [56]. Out of the total of 523 autopsies, subjects were selected only if the following criteria were satisfied:

1. Subjects with complete physical, neurologic, and cognitive assessment in an interval prior to death (Cog-Death interval) of ≤1.0 year.
2. Neuropathologic assessments including CERAD pathologic diagnostic and Braak-NFT scores
3. Availability of APOE genotype.

Subjects were excluded if:

1. Met the criteria for mixed dementia. This exclusion criteria was particularly important to minimize the influence of cerebro-vascular pathologies on the cognitive deficits.
2. Met the criteria for dementia with Lewy bodies, Parkinson's disease with dementia, Pick's disease, Creutzfeldt-Jacob disease.
3. Showed hippocampal sclerosis, tauopathies, or primary or secondary brain tumors.

Based on the clinical, cognitive, and neuropathologic assessment and above described inclusion/exclusion criteria, we identified a total of 155 subjects suitable for this study. These subjects were assigned to four study groups:

1. Age-matched controls (C): subjects with intact cognition and no significant AD pathology at autopsy (CERAD = 0; Braak 0-II) (*n* = 11)
2. Asymptomatic AD (ASYMAD): subjects with intact cognition and AD pathology at autopsy (CERAD: B-C; Braak 0-VI) (*n* = 13)
3. Mild cognitive impairment (MCI): subjects with MCI diagnosis and AD pathology at autopsy (CERAD: B-C; Braak: 0-VI) (*n* = 15)
4. Alzheimer's disease (AD), patients with clinical dementia and diagnosis of definite AD at autopsy (CERAD: B-C; Braak: 0-VI) (*n* = 116).

We decided not to classify these cases with neither NIA-Reagan criteria [57] nor AA-NIA criteria [58]. The reasons for this were:

1. NIA-Reagan criteria apply to autopsy cases with previously ascertained dementia, then *per se*, not applicable to C or ASYMAD
2. AA-NIA pathologic criteria do not take into account the diagnosis of dementia to stage neuropathologic AD changes, so excluding an important aim of our study: analyzing the presence or absence of cognitive impairment as related to AD pathology, APOE genotype and educational levels.

Moreover, NIA-Reagan and AA-NIA criteria are probabilistic systems of classification relying on A*β*-NP and NFT scores with the assumption that a cumulative pathogenetic contribution to AD pathogenesis is actually originating from both types of pathology. This assumption has not been demonstrated yet. Although NIA-Reagan and AA-NIA criteria have an undoubted practical utility in a clinical setting, in this investigation we preferred to consider CERAD and Braak scores separately. This choice was also useful to verify possible distinct cognitive-pathologic-genotype correlations as subjacent to different pathogenetic mechanisms, such as A*β*-NP or NFT formation.

### APOE genotyping

APOE genotype analyses on all study subjects have been conducted on DNA isolated from buccal swabs (archival) or from brain tissues as previously described [59].

### Statistical analyses

ANOVA was used to compare continuous variables (i.e. mean age at death, mean age at last cognitive examination before death, mean score of the last MMSE, mean scores of ADLs, and mean brain weight [BW]) across all groups; while Fisher's exact test was used to compare categorical variables (i.e. CERAD pathological scores, Braak-NFT staging, and educational levels attained).

For APOE allelic frequency analyses, we used Fisher's exact test for a 3-by-4 contingency table comparing the frequencies of all three APOE allele across all groups. Then, we carried out analyses to examine the hypothesis that APOε2 was enriched in ASYMAD by conducting additional Fisher's exact test to compare the frequencies of APOε2 vs. all other alleles in ASYMAD vs MCI, AD, and C, separately.

In addition, an exact Fisher's test was performed to compare the APOE allelic frequencies in two larger combined groups: 1) subjects with cognitive deficits (MCI+AD) (impaired cognition group, ICG); and 2) subjects without cognitive deficits (C+ASYMAD) (preserved cognition group, PCG). This analysis was performed to investigate possible effects of APOε2 exclusively based on the documented cognitive assessment shortly before death. Separate analyses using an exact Fisher's test were performed to compare ICG and PCG on the highest educational level attained. Finally Spearman's test for nonparametric data (APOε2 frequencies) and highest level of education attained (master or higher) across all groups, expressed as percentages, was also performed to test if significant correlation between APOε2 and higher education was possibly present.
